# Digital surgical planning and placement of osseointegrated implants to retain an auricular prosthesis using implant software with cone‐beam computed tomography and 3D‐printed surgical guides: A case report

**DOI:** 10.1002/ccr3.3499

**Published:** 2020-11-11

**Authors:** Daniel Domingue, Naif Sinada, James R. White

**Affiliations:** ^1^ Drs. Smith and Domingue Lafayette LA USA; ^2^ Private Practice Fayetteville AR USA; ^3^ Private Practice Lafayette LA USA

**Keywords:** 3D printing, auricular prosthesis, cone‐beam computerized tomography, osseointegrated implants, surgical guide

## Abstract

Integration of CBCT imaging with dental implant treatment planning software and 3D‐printed surgical guides can facilitate surgical planning for extraoral implant placement. In the current case, this combined planning strategy enabled navigation of challenging osseous anatomy, avoided critical structures, and optimized osseointegration for prosthesis retention.

## Graphical abstract

Integration of CBCT imaging with dental implant treatment‐planning software and 3D‐printed surgical guides can facilitate surgical planning for extraoral implant placement in challenging osseous anatomy to retain an auricular prosthesis.

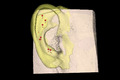

## 1 INTRODUCTION

Improvements in reliable auricular prosthetic replacement^1^ necessitated by cancer,^2^ trauma (including burns^3^),^4^, ^5^, ^6^ are increasingly in demand. Both acceptable^1^, ^13^ and suboptimal outcomes have been reported.^6^

Conventional analog approaches used to fabricate implant‐retained auricular prostheses involve making a conventional impression of the affected and unaffected ear sites (using the intact ear as an indirect template),^7^, ^14^ fabrication of a gypsum‐product master cast,^7^, ^15^, ^16^, ^17^ and production of a wax pattern,^7^, ^14^, ^17^ used to fabricate the definitive prosthesis via conventional flasking, wax‐elimination, and molding and curing of the prosthesis, commonly using silicone. Various modifications of this conventional technique have been described with the objective of overcoming technique‐sensitivity hurdles, such as tissue movement, ^16^ accurate reproduction of convoluted auricular anatomic dimensions with severe undercuts,^14^ and impression distortion.^17^, ^18^ Acquisition of similar ear anatomy via an impression from an individual other than the patient to create a wax pattern for fabricating the prosthesis has also been described.^14^

The co‐evolution of digital planning and design with temporally placed osseointegrated implants has improved auricular replacement workflows and outcomes.^4^, ^5^, ^6^, ^10^, ^11^, ^19^ Twenty‐year survival rates of over 97% were reported in a retrospective analysis by Subramaniam et al.^11^ of 341 temporal osseointegrated implants placed in 110 patients. Extraoral craniofacial implants placed in patients with histories of head‐and‐neck oncologic surgery or radiation therapy have consistently higher failure rates.^2^, ^6^, ^11^ Another retrospective survival analysis by Curi et al.^5^ reported a 2‐year survival rate of 94.1% for auricular implants in craniofacial rehabilitation patients, some of whom had irradiation histories. Woods and Chandu^6^ reported an auricular implant failure rate of 17% in head‐and‐neck surgery patients. Computed tomography (CT)‐driven preoperative planning in osseointegrated implant‐retained auricular replacement procedures is being actively reported.^2^, ^10^, ^12^, ^13^, ^19^, ^20^ This clinical report focuses on digital guidance parameters for strategic placement of osseointegrated implants in sound temporal bone using three‐dimensional (3D) cone‐beam computed tomography (CBCT) planning as the first stage of auricular replacement, to optimize effective implant support for the final prosthesis.

A 54‐year‐old Caucasian man was missing his right ear due to a work‐related accident. After injury, a nonviable pinna remnant necessitated a complete auriculectomy but his tragus remained intact. He had a normal external auditory meatus, external auditory canal, and tympanic membrane; an audiologic workup revealed normal hearing in this ear. His medical history was otherwise unremarkable, he had no known drug allergies, was on no blood‐thinning medications, and had no radiotherapy or chemotherapy history. The patient provided written informed consent in accordance with the currently amended Declaration of Helsinki.

Key features of the surgical phase of this auricular replacement included: 1) optical scanning of the contralateral (left) ear; 2) 3D cone‐beam computed tomographic (CBCT) imaging of the mastoid portion of the right temporal bone using compatible open‐source implant treatment‐planning software; 3) two 3D‐printed custom stereolithographic surgical guides, for implant placement.

A CBCT scan of the skull (PreXion 3D cone beam, PreXion, Inc) and optical scan of the contralateral ear (3Shape A/S) were obtained. The optical scan image was duplicated, mirrored, and a solid base added to convert it into a printable right ear using open‐source computer‐aided design (CAD) software (Meshmixer, Autodesk Research). Meshmixer is a triangle‐mesh‐based software capable of importing and exporting in stereolithography (STL) file format, which has a high level of compatibility with 3D surgical planning and 3D printing applications.

The converted STL file of the planned prosthesis was imported and integrated into a Digital Imaging and Communications in Medicine (DICOM) file in the planning software (Blue Sky Plan, Blue Sky Bio, LLC). Blue Sky Plan is an advanced treatment‐planning software application for computer‐guided implant‐placement surgery, with the capability to directly import patient CBCT scan data and export STL files from the software for 3D printing of surgical guides.

**FIGURE 1:**
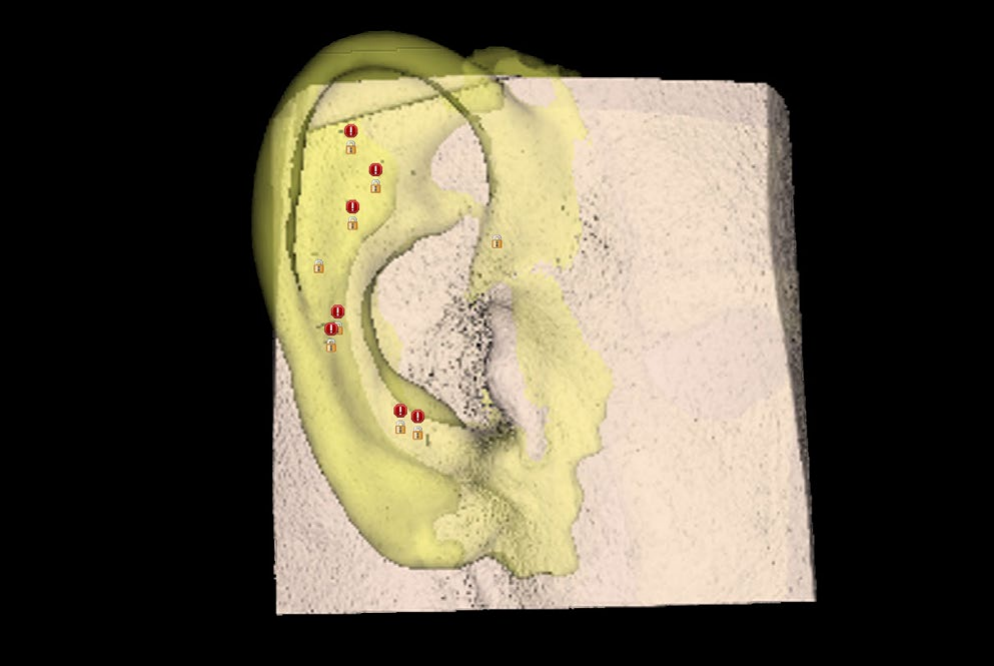
Planned prosthetic right ear and soft tissue. Red markings are produced by planning software (Blue Sky Plan, BlueSky Bio, LLC) to indicate potential problem areas for implant placement

**FIGURE 2:**
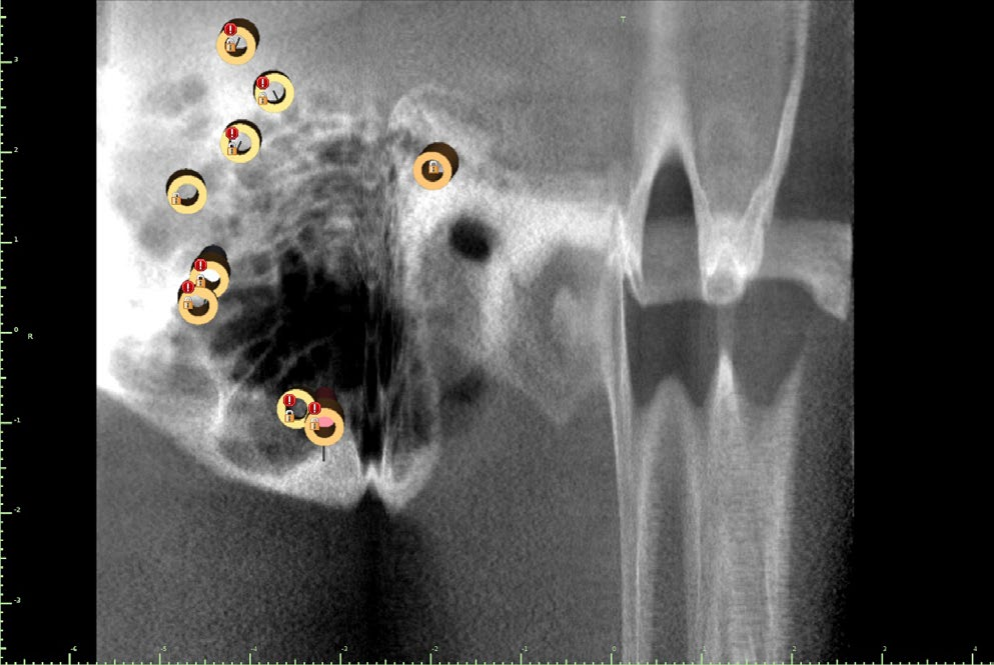
Axial CBCT image of prospective implant sites on right temporal bone, showing significantly pneumatized mastoid portion

The DICOM file was converted into a soft‐tissue contour and the imported STL file of the inverted contralateral ear virtually overlaid, as seen in Figure 1. Integration of these data enabled plotting and viewing of optimal available bone quantity and quality for implant placement and restorability relative to predetermined prosthesis attachments. The intention was to place as many implants as possible, to provide fallback placement options intraoperatively. Eight custom titanium implants (Vistafix, Cochlear, Ltd.) were planned for placement into the right temporal bone, avoiding critical structures (thin calvarium wall, dura mater, mastoid air cells), as seen on axial CBCT in Figure 2.

**FIGURE 3:**
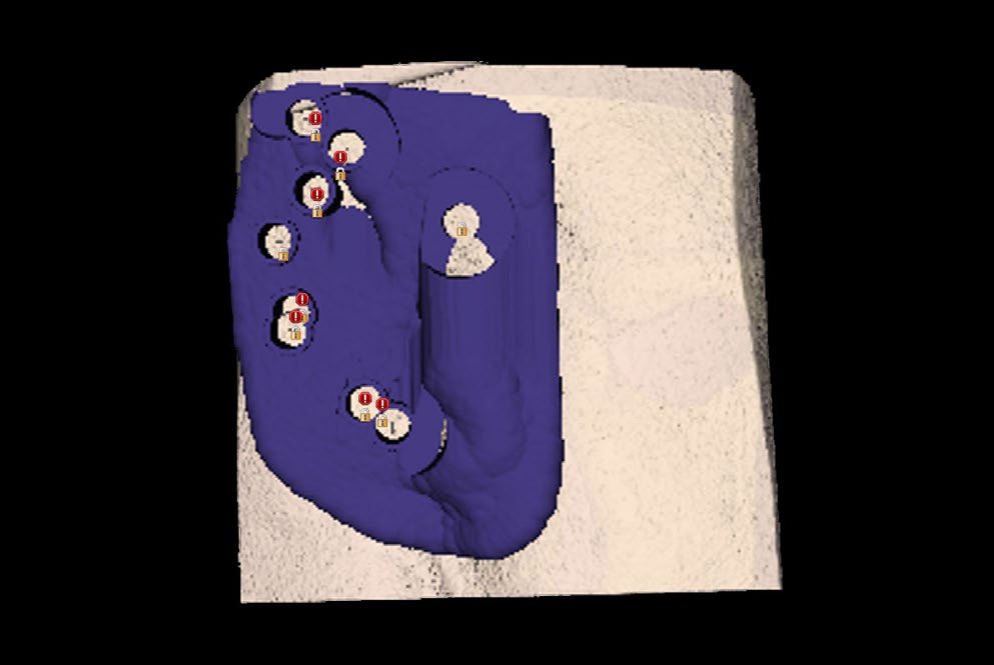
Soft‐tissue prosthetic guide (Blue Sky Plan, BlueSky Bio, LLC)

**FIGURE 4:**
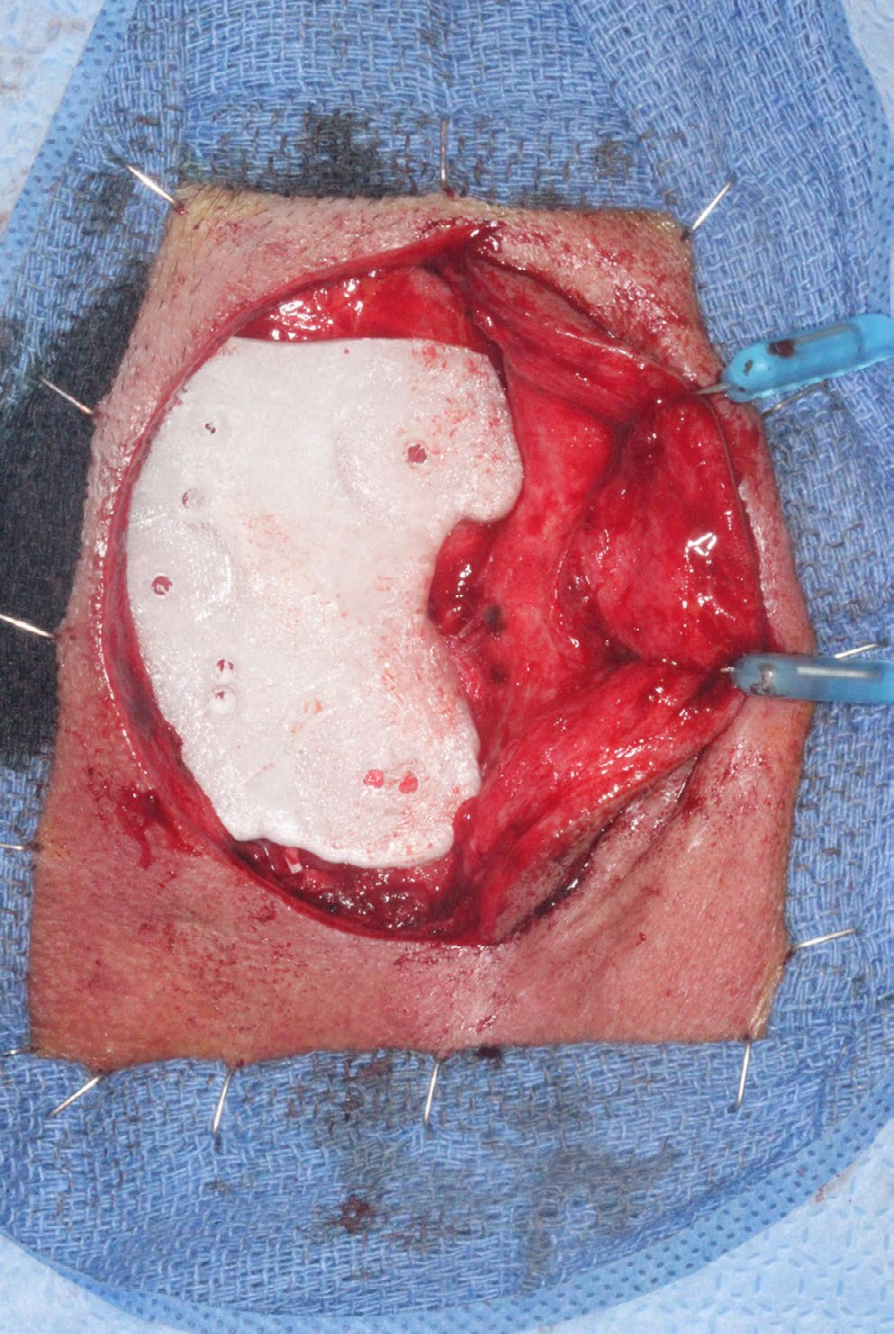
Bone surgical guide showing eight planned osteotomy sites (Blue Sky Plan, BlueSky Bio, LLC)

**FIGURE 5:**
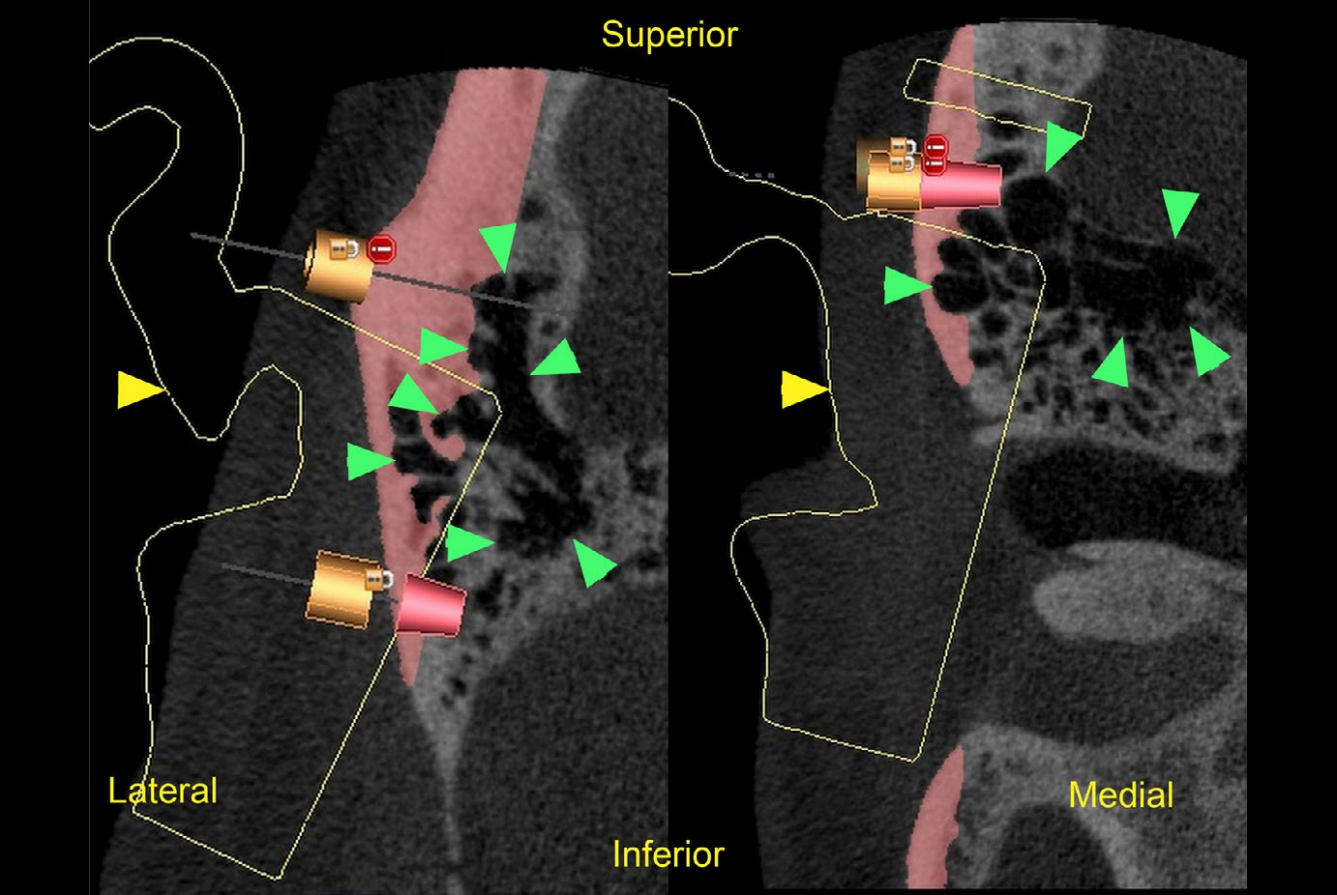
DICOM imaging slices showing preoperative implant planning; planned prosthetic ear (yellow outline, yellow arrow); temporal bone (note mastoid air cells, green arrows). Image oriented to patient sitting upright

A prosthetic soft‐tissue guide was 3D‐printed using CBCT‐generated bony topographic scan data, ensuring proper alignment between planned implant and prosthesis positions, as seen in Figure 3. As seen in Figure 4, a bone‐supported surgical guide was also 3D‐printed using the DICOM data, rendered in sagittal, coronal, and axial views, as seen in Figure 5, to guide angulation, depth, and position. The guides and prosthesis were cold‐sterilized for intraoperative use. The guide sleeve tubes were customized to fit the pilot drill. Both guides and prosthetic ear were 3D‐printed on a CEL Robox 3D in‐office printer (C Enterprise Ltd.[CEL]) from nGen material (ColorFabb BV).

Profound local anesthesia was obtained at the surgical site with 1% lidocaine with 1:100 000 epinephrine. General endotracheal anesthesia was obtained and 2 g of intravenous cefazolin administered. Attachment sites were marked on the skin through the soft‐tissue guide.

A standard “C”‐shaped post‐auricular incision was made to the periosteum with a No. 15 blade. The skin and subcutaneous tissues were elevated anteriorly to the external auditory canal, leaving the periosteum and temporalis fascia intact, allowing the bone surgical guide to seat completely on the periosteum. All remaining potential osteotomy sites were then marked on the periosteum, through the bone guide, as seen in Figure 4. The periosteum was reflected off the bone at each site, the guide removed, and four osteotomies drilled using copious sterile saline irrigation, at a drill speed of 2000 rpm.

**FIGURE 6:**
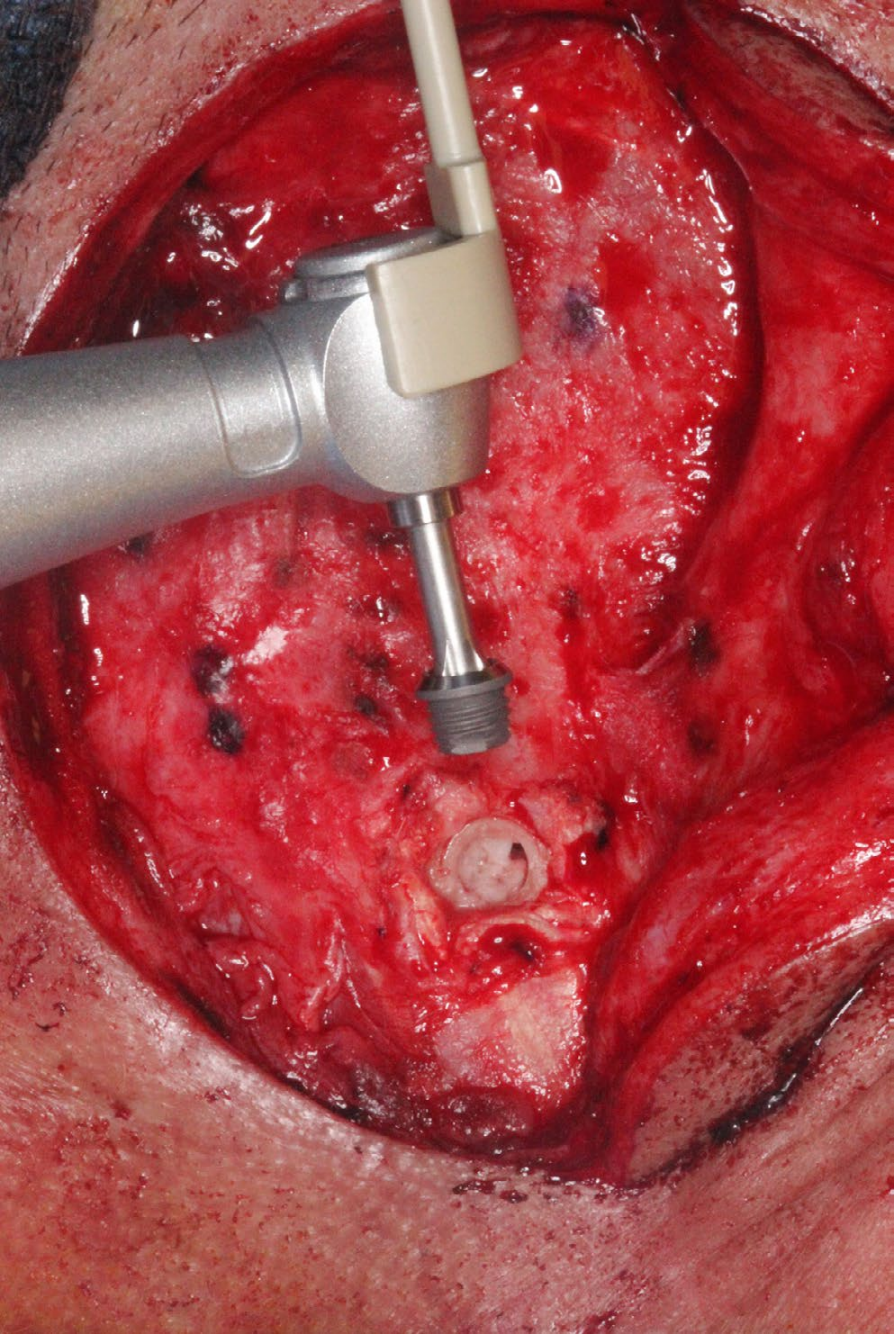
One of four osteotomy sites, after reflection of periosteum at that site, shown with 4.5‐mm diameter × 3‐mm length implant, before placement

**FIGURE 7:**
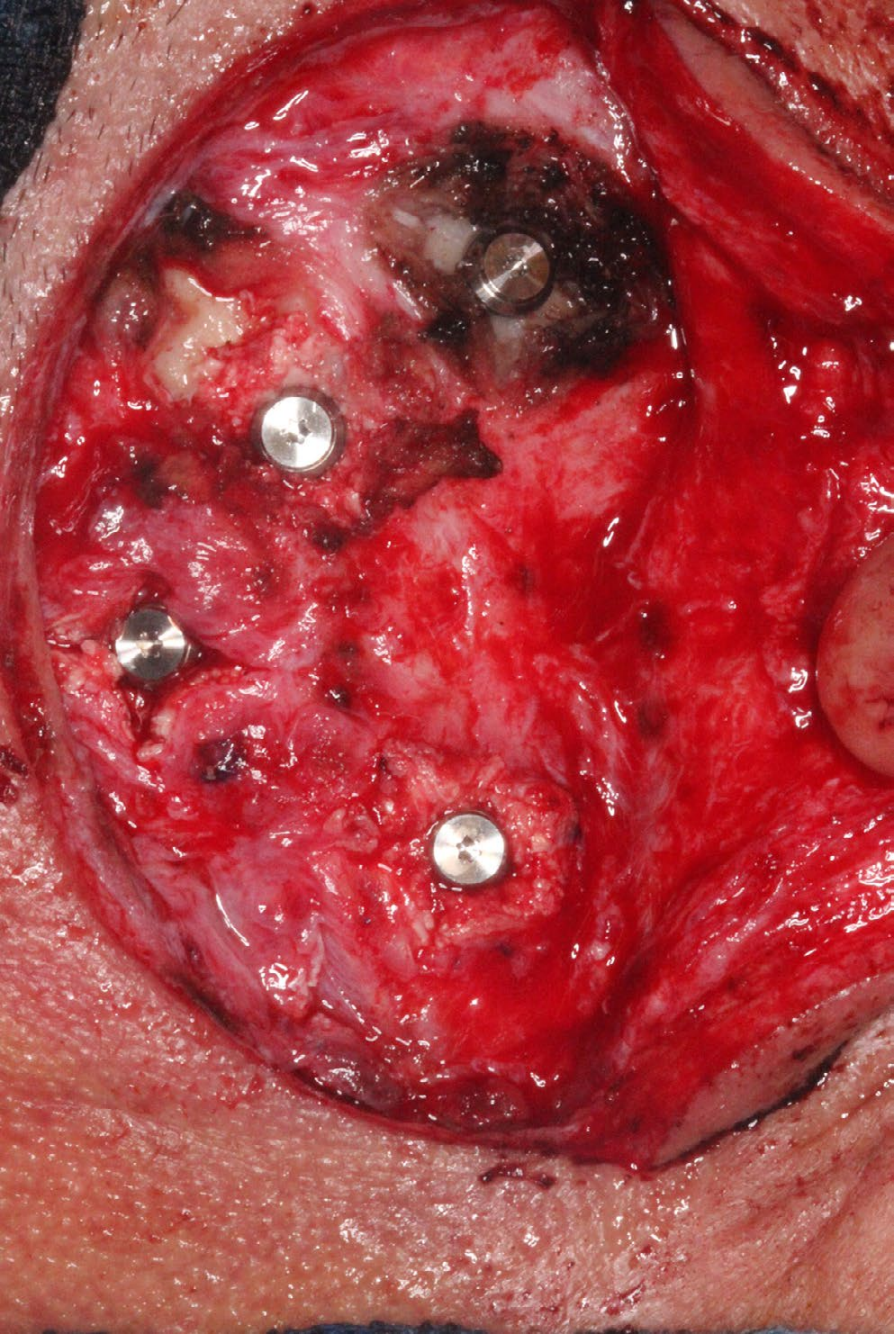
Four implants (two 3‐mm length; two 4‐mm length) placed, with cover screws

**FIGURE 8:**
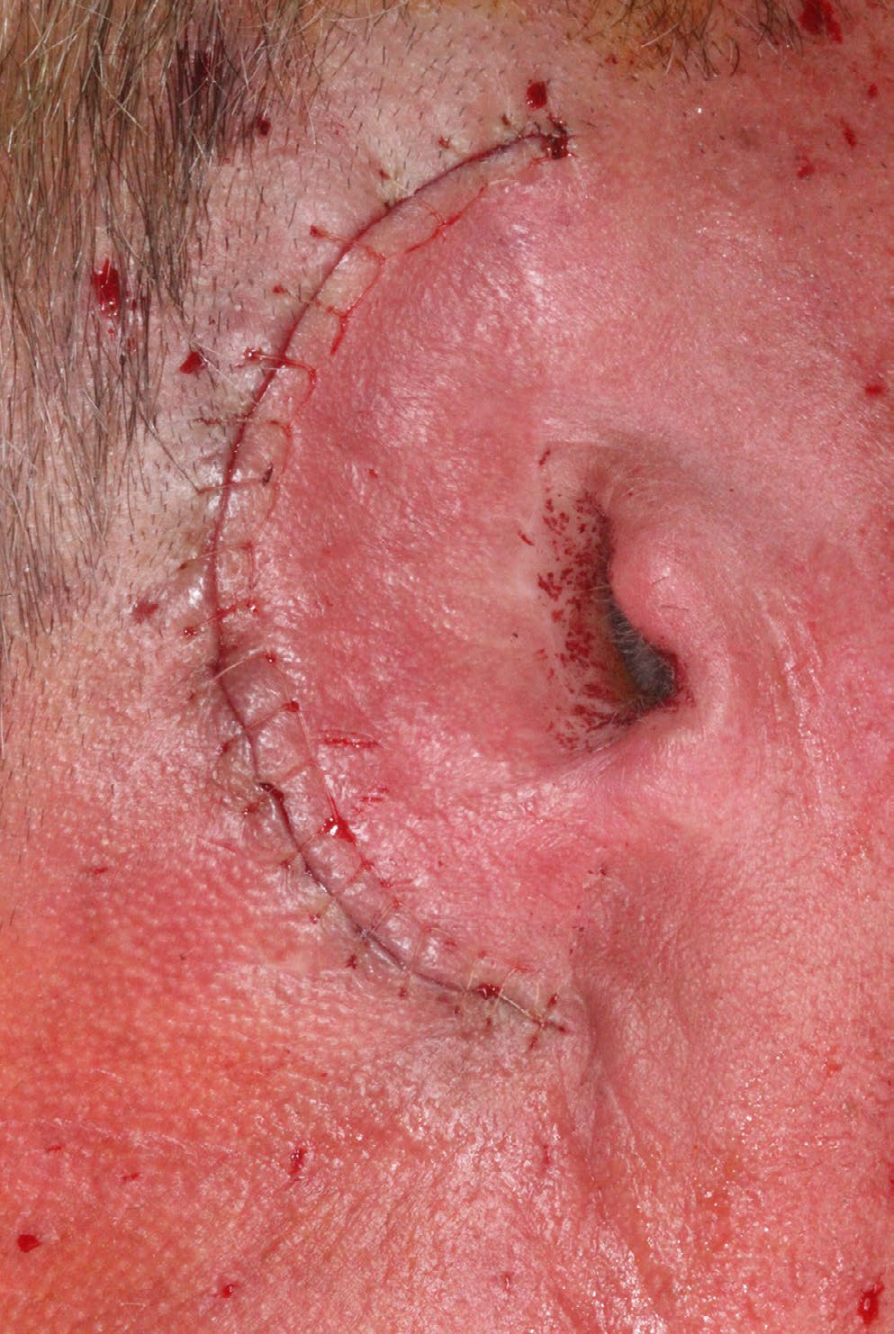
Surgical site after completion of primary closure (5‐0 plain gut sutures used for skin closure)

The first completed osteotomy site and implant (before placement) are seen in Figure 6. Four implants were definitively placed, as seen in Figure 7. All were 4.5 mm in diameter; two were 3 mm and two 4 mm long. All implant fixtures were torqued to at least to 30 Ncm. Three of the four sites were grafted with osteotomy‐generated autogenous bone. After irrigation with bacitracin in normal saline, tension‐free primary closure was obtained with subcutaneous 3‐0 polyglactin 910 (Vicryl, Ethicon US, LLC) and superficial skin sutures (5‐0 fast‐absorbing plain gut), as seen in Figure 8, allowing a 14‐week osseointegration period.

Detailed CBCT scans of the patient's right temporal bone identified a significantly pneumatized mastoid portion, as seen in Figures 2 and 5, which limited the number of stable implant sites that could be esthetically concealed beneath the antihelix portion of the prosthesis. Without this 3D information, selection of more “typical” osteotomy sites may have involved a very thin cortex of bone within these mastoid air cells, possibly jeopardizing osseointegration. For the current treatment, CBCT facilitated capture of greater osseous detail than could conventional CT; this, in turn, facilitated contingency planning of eight viable sites, only four of which were used.

**FIGURE 9:**
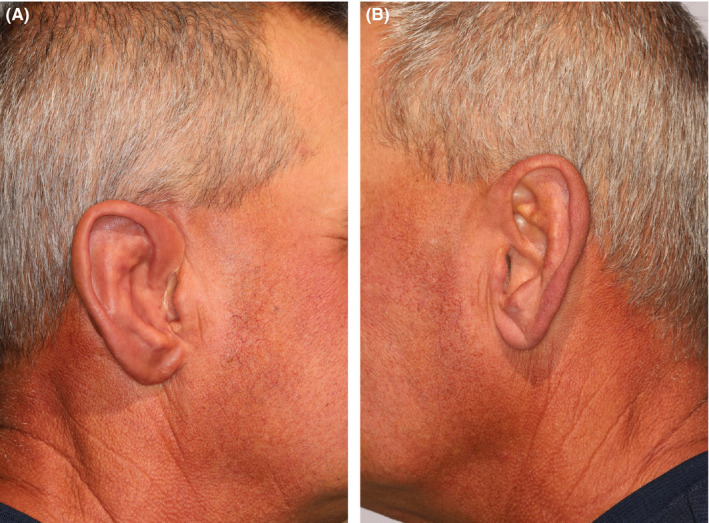
A,Completed prosthesis in place (fabrication described elsewhere [In press; Ref.^21^]). B, Contralateral ear, used for fabricating prosthesis (fabrication described elsewhere In press; Ref.^21^])

**FIGURE 10:**
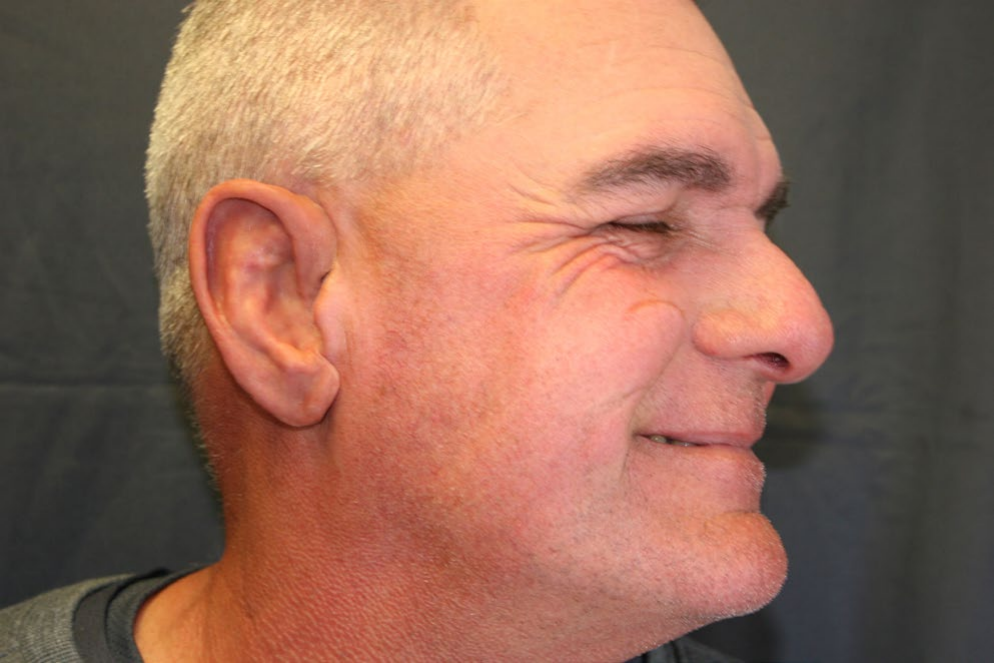
Prosthesis in place in patient (fabrication described elsewhere In press; Ref.^21^])

For the final prosthesis, three of these four implants were fitted with abutments and attachments, and provided a structurally stable and highly esthetic result (as seen if Figure 9A and Figure 10), described in detail elsewhere. [Domingue et al.]^21^

The 3D‐printed surgical guides and CBCT guidance enabled osteotomy and implant placement in sound bone, avoiding intraoperative complications such as bleeding, inner cortical perforations of the pneumatized mastoid portion into underlying tissues, angulation discrepancies, or compromised osseointegration, thus optimizing a prosthetically driven approach to the surgical phase, and in turn, also optimizing the final prosthetic result, seen in Figure 9A (with contralateral ear, seen in Figure 9B) and Figure 10 (patient's right profile with prosthesis in place) and described in detail elsewhere. [Domingue et al. In press.]^21^

## 2 DISCUSSION

Implant‐retained auricular prostheses have a considerable track record of success in the literature, beginning with a 38‐patient retrospective series by Tjellstrom et al. in (1985)^22^ and including others dating to 2002.^1^, ^4^, ^5^ Of note, a 25‐year retrospective analysis of 341 craniofacial implants in 110 patients by Subramaniam et al.^11^, temporal implants overall showed the highest comparative success rate (97%) and statistically significant) prosthetic (*P* < .0001) and implant survival rates (*P* < .0001). Brandao et al.^4^ also reported an auricular implant success rate of over 98%; two‐implant retention showed the lowest failure rate. While adhesive‐ and bar‐retained prostheses have shown clinical success,^7^, ^23^, ^24^ a 2012 review by Sharma et al.^25^ identified benefits of implant retention versus adhesive. While approaches similar to the current situation (scanning, digitization and 3D‐printing) have been reported,^9^, ^10^, ^12^, ^13^, ^26^ all used conventional CT^9^, ^10^, ^13^, ^26^ or electron‐beam tomography.^12^ The current treatment used CBCT with specific implant‐planning software, which offered the surgical advantages of (a) direct 3D printing of the surgical guides and (b) CBCT‐generated data reflecting the patient's anatomy at multiple levels. To date, CBCT use in treatment planning for auricular prostheses has been limited to implant placement in cadaver skulls^27^and has limited reported clinical use.^28^ To the authors' knowledge, this is the first clinical report to describe 3D CBCT use for both (a) assessment of soft‐tissue and temporal bony topography *and* (b) 3D‐printing of soft‐tissue *and* bone surgical guides for an auricular prosthesis. Clinically reliable auricular replacement has been reported as early as 2002.^1^, ^2^, ^3^, ^4^, ^5^, ^6^, ^13^ More prospective studies assessing multilevel 3D anatomic visualization are needed. Finally, increased interdisciplinary collaboration by teams comprising dental implantologists and otolaryngologic surgeons (as demonstrated in this patient's treatment and in a 2014 report by Felisati et al.) is also critically needed.^29^

## CONFLICT OF INTEREST

None of the authors listed on this article has any potential conflict of interest, financial or otherwise, with any entity, product, or aspect of any treatment described in this article.

## AUTHOR CONTRIBUTION

Daniel Domingue, DDS (DD): Developed and oversaw patient's treatment plan (patient of record in DD's restorative/implant dental practice); designed surgical guides, collaborated with and shared data with NS; took photos of patient; produced initial draft of manuscript; critically reviewed and provided input on all drafts and approved final version of article manuscript. Naif Sinada, DMD, MS, MPH (NS): Provided DD with concept of digital design and construction of the replacement ear against a backdrop of analog fabrication methods (based on training and expertise in maxillofacial prosthetics); developed and refined process based on DD's data; consulted with DD throughout the patient's treatment; critically reviewed and provided input on all drafts and approved final version of article manuscript. James R. White, Jr, MD (JRW): Developed surgical treatment plan in collaboration with DD; performed implant placement surgery on patient in the operating room; drafted manuscript text to describe surgical implant‐placement procedure; critically reviewed and provided input on all drafts and approved final version of article manuscript.

## ETHICAL APPROVAL

There was no formal ethics committee approval for the current study, as it is a single patient case in a private dental practice. The patient provided signed informed consent to undergo the procedure, in accordance with the currently amended Declaration of Helsinki.
